# Evidence for henipavirus spillover into human populations in Africa

**DOI:** 10.1038/ncomms6342

**Published:** 2014-11-18

**Authors:** Olivier Pernet, Bradley S. Schneider, Shannon M. Beaty, Matthew LeBreton, Tatyana E. Yun, Arnold Park, Trevor T. Zachariah, Thomas A. Bowden, Peta Hitchens, Christina M. Ramirez, Peter Daszak, Jonna Mazet, Alexander N. Freiberg, Nathan D. Wolfe, Benhur Lee

**Affiliations:** 1 Department of Microbiology, Immunology and Molecular Genetics, David Geffen School of Medicine at UCLA, Los Angeles, California 90095, USA; 2 Global Viral/Metabiota Laboratory Sciences, San Francisco, California 90104, USA; 3 Department of Microbiology, Icahn School of Medicine at Mount Sinai, New York, New York 10029, USA; 4 Department of Pathology, University of Texas Medical Branch, Galveston, Texas 77555, USA; 5 Brevard Zoo Veterinary Services, Brevard Zoo, Melbourne, 32940 Florida, USA; 6 Division of Structural Biology, Wellcome Trust Centre for Human Genetics, University of Oxford, Oxford, OX3 7BN UK; 7 Department of Medicine and Epidemiology, School of Veterinary Medicine, UC Davis, Davis, California 95616, USA; 8 Department of Biostatistics, School of Public Health, UCLA, Los Angeles, California 90095, USA; 9 EcoHealth Alliance, New York, New York 10001, USA

## Abstract

Zoonotic transmission of lethal henipaviruses (HNVs) from their natural fruit bat reservoirs to humans has only been reported in Australia and South/Southeast Asia. However, a recent study discovered numerous HNV clades in African bat samples. To determine the potential for HNV spillover events among humans in Africa, here we examine well-curated sets of bat (*Eidolon helvum, n*=44) and human (*n*=497) serum samples from Cameroon for Nipah virus (NiV) cross-neutralizing antibodies (NiV-X-Nabs). Using a vesicular stomatitis virus (VSV)-based pseudoparticle seroneutralization assay, we detect NiV-X-Nabs in 48% and 3–4% of the bat and human samples, respectively. Seropositive human samples are found almost exclusively in individuals who reported butchering bats for bushmeat. Seropositive human sera also neutralize Hendra virus and Gh-M74a (an African HNV) pseudoparticles, as well as live NiV. Butchering bat meat and living in areas undergoing deforestation are the most significant risk factors associated with seropositivity. Evidence for HNV spillover events warrants increased surveillance efforts.

## Abstract (web summary)

Henipaviruses (HNVs) infect bats in Asia and Africa, but transmission to humans (often with lethal consequences) is known only in Asia. Here the authors show that 3% of human serum samples from certain areas in Cameroon contain antibodies against HNV, indicating spillover into the human population.

## Graphical abstract

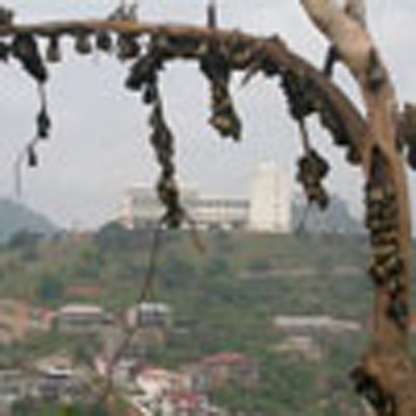

Nipah (NiV) and Hendra viruses (HeV) are highly pathogenic paramyxoviruses of the henipavirus (HNV) genus that cause acute encephalitis and respiratory illness. Their mortality rate in humans can be greater than 90% (refs 1, 2) and they are the only paramyxoviruses that are classified as biosafety level 4 (BSL-4) pathogens. Until recently, the *Henipavirus* genus contained only two species—HeV and NiV viruses—which are phylogenetically closely related and exhibit serological cross-reactivity^3^. Fruit bats within the suborder *Megachiroptera*, particularly those of the genus *Pteropus,* have been identified as the natural reservoir for HNVs^4^,^5^,^6^,^7^. The geographical distribution of these reservoir bats partially coincides with the distribution of HNV outbreaks and spillover events around the Indian Ocean, reaching from Australia (HeV) to Southeast Asia and the Indian subcontinent (NiV). Ecological studies^8^ have revealed several characteristics common to all regions of HNV outbreaks: (i) they are the natural habitat of Pteropid bats (*Pteropus* spp.), (ii) bat habitats in the region have been dramatically altered by the introduction of domestic plant and animal species and concomitant deforestation of the natural landscape^9^ and (iii) humans or domestic animals have direct contact with bats in the area. Although these characteristics can be observed in other locations around the world, to date, HNV outbreaks and spillovers into human populations have only been recognized in Australia and South Asia.

The geographic distribution of *Pteropus* and other Pteropodids (Old World fruit bats) extends well beyond areas with documented HeV and NiV outbreaks. In 2007, a survey of Pteropodid species in Madagascar^10^ reported that 2.3% and 19.2% of serum samples from *Pteropus rufus* and *Eidolon dupreanum*, respectively, tested positive for cross-reactive anti-HNV antibodies. Malagasy fruit bats share ecological niches, either roosting in the same caves or feeding in the same fruit trees. As *Eidolon* species are extremely mobile (they can fly up to 2,500 km per year^11^,^12^) and are present all around sub-Saharan Africa, Iehlé *et al.* raised the possibility of lateral transfer of HNV from or to other *Eidolon* species on mainland Africa and hypothesized a much wider distribution of HNV^10^. Indeed, anti-HNV antibodies were soon found in *E. helvum* (the common straw-coloured African fruit bat) from Ghana^13^ on the west coast of Africa, and more recently on Annobón island^14^ in the Gulf of Guinea. Furthermore, HNV-like RNA sequences have been identified in faecal droppings of urban roosting bats in Ghana^15^, and more ominously, in fruit bat bushmeat in the Republic of Congo^16^.

Recently, sequence analysis of a larger sample set collected from western and southern Africa revealed a surprising diversity of paramyxoviruses in African bats, including 19 new species of HNV-like viruses distinct from the Nipah and Hendra viruses found in Southeast Asia and Australia^17^. However, only one almost complete African HNV-like genome sequence (Gh-M74a clone) has been published to date, and the corresponding viral isolate has not been reported. This sequence was derived from a bat specimen originating in Ghana. We will refer to this putative HNV-like virus as the Ghana virus (GhV), and GhV-F and GhV-G when referring to its fusion (F) and attachment (G) envelope glycoproteins, respectively. In contrast to the 80–90% sequence identity shared between the F and G envelope glycoproteins of NiV and HeV, GhV-F and GhV-G share only about 70 and 40% sequence homology and even lower sequence identity (56 and 26%) with their respective NiV and HeV counterparts. Given this poor overall sequence conservation, it is unclear whether humoral responses elicited against the F/G proteins from African clades of HNV-like viruses would cross-react with F/G from NiV or HeV. This sequence divergence highlights the limitations faced by current seroprevalence studies that rely mostly on ELISA- or Luminex-based assays using recombinant NiV-G or HeV-G proteins as the target antigen^10^,^13^,^14^,^18^.

ELISA-based screening assays, although efficient, can yield high false positive and false negative rates compared with functional seroneutralization (SN) assays^19^. Thus, whenever possible, ELISA/Luminex-positive samples are confirmed by a SN assay. Although SN assays are considered a gold standard for seroprevalence studies^19^,^20^,^21^, follow-up confirmation with live virus SN assays is limited by the amount of sample available, and the requirement to work with live HNV in a high-containment facility (BSL-4). Consequently, in many prior studies only ELISA/Luminex-positive samples, and often only a small subset such as those with the highest binding activity, were confirmed with a biological or surrogate SN assay (reviewed in LF Wang *et al.*^21^; for example, AJ Peel *et al.*^22^). The latter is based on serum antibody competition of soluble receptor (sEphrinB2-Fc) binding to recombinant NiV-G or HeV-G conjugated to Luminex beads^18^. Although these procedures can guard against false positives, they do not address the loss of potential false negatives^10^,^13^,^14^.

Given the recent reports that a diversity of HNV-like viruses are present and may be widely distributed in the bat reservoir host population across Africa^17^,^22^, in the present study, we sought to evaluate the seroprevalence of HNV-like infections in both the bat and proximate human populations in Cameroon, and to assess risk factors that might be associated with any putative zoonotic transmission of African HNV-like viruses. To avoid the specificity and sensitivity issues associated with ELISA-based assays, as well as the impracticalities of using a live virus SN assay in BSL-4 as a screening test, we developed a VSV-based HNV envelope pseudotype particle (VSV-HNVpp) infectious SN assay^20^, which can be used at BSL-2 conditions as a primary screen for anti-NiV cross-neutralizing antibodies (anti-NiV-X-Nabs). Wang and Daniels raised the possibility that the high sensitivity and specificity of the pseudotyped particle platform may allow for the combination of screening and confirmatory tests in a single assay^21^. Thus, we screened serum samples from hunted bats (*E. helvum*) in an urban area in Yaoundé, Cameroon, and from almost 500 humans living in various villages across the south of Cameroon. The specificity, breath and potency of anti-NiV-X-Nabs were confirmed using numerous specificity controls unique to our infectious SN assay, including isogenic viruses pseudotyped with irrelevant (VSV-G) or related HNV envelopes (HeV and GhV), and follow-up confirmation with a recombinant replication-competent reporter NiV specifically engineered for high-sensitivity detection of anti-NiV-X-Nabs. Remarkably, the seropositive human samples were found almost exclusively in individuals who reported butchering bats for bushmeat. The geographical and temporal clustering of these seropositive cases provides evidence for recent HNV-like spillover events into the human population in this part of Africa.

## Results

### NiV cross-neutralizing activity in bat serum samples

**Figure 1:**
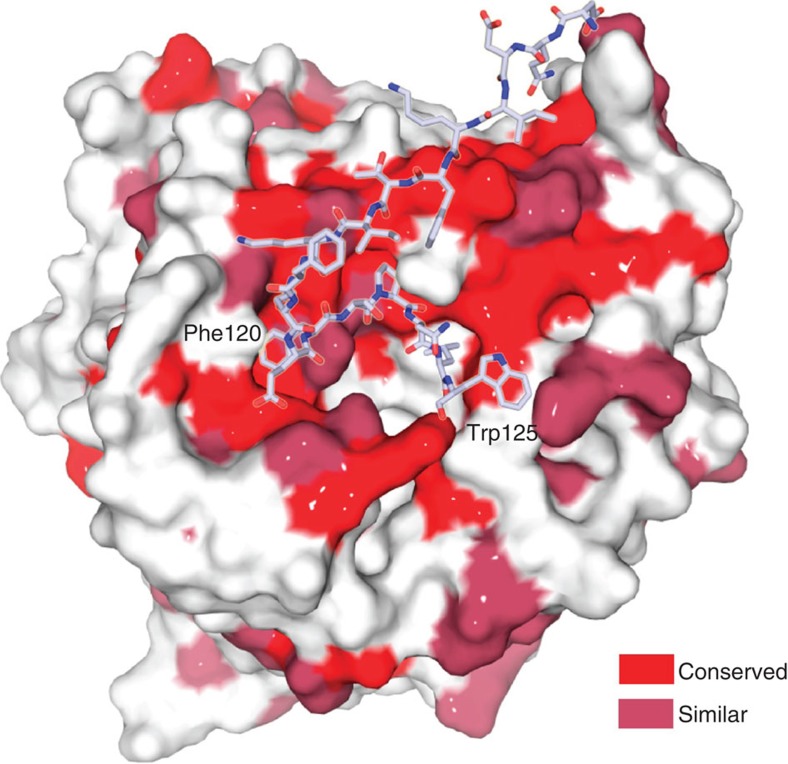
Mapping sequence conservation of GH-M74a onto the surface of NiV-G. NiV-G surface is coloured according to residue conservation with GH-M74a: red, conserved; maroon, similar; white, no sequence conservation. EphrinB2 residues 107–127 are represented as sticks. Although NiV-G shares relatively low sequence conservation with GhV-G (approximately 25%), it shares greater sequence conservation (45%) in residues that make up the receptor-binding site (calculation performed with PISA EBI server).

Despite the overall low sequence homology between GhV-G and NiV-G/HeV-G, mapping of the GhV-G sequence onto the crystal structure of NiV-G complexed with ephrinB2 (ref. 23) indicated that the vast majority of the sequence conservation was located at the receptor-binding interface, suggesting that GhV-G may also use ephrinB2 (and likely ephrinB3) as receptors for cell entry (Fig. 1). The clustering of conserved sequences around the receptor-binding site raises the possibility of biologically significant anti-NiV *cross-neutralizing* antibodies (anti-NiV-X-Nabs) in African bats exposed to African clades of HNV-like viruses despite their overall low sequence identity with NiV. To determine the prevalence of anti-NiV-X-Nabs in that geographically proximate part of Western Africa, we screened fruit bat (*E. helvum*) serum samples from Cameroon, collected and curated by Global Viral/Metabiota. To conserve the use of such sera, we further optimized a previously validated VSV-based (VSV-ΔG-rLuc) NiV envelope pseudotyped particle (NiVpp) SN assay for high-specificity screening, and established appropriate control sera as described in Methods (Supplementary Fig. 1).

**Figure 2:**
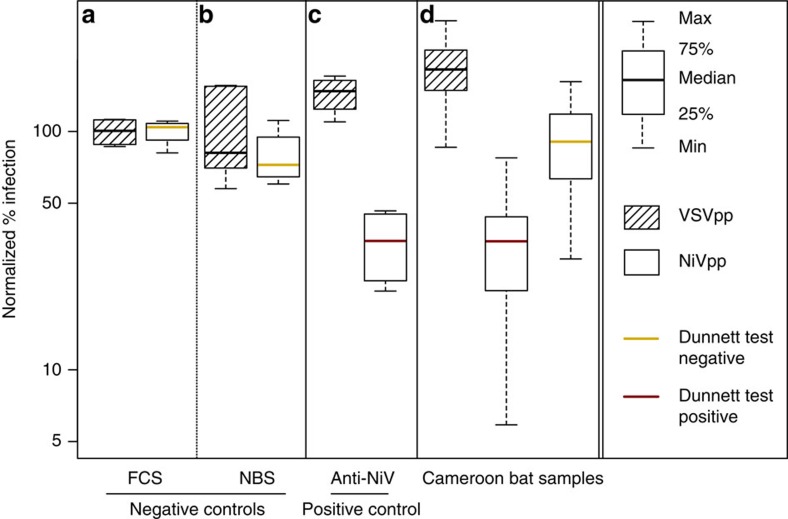
Prevalence of anti-NiV cross-neutralizing antibodies in bat sera from Cameroon. Box-and-whisker plots showing the infection (normalized to the negative control fetal calf serum, FCS) of Vero cells by VSVpp (isogenic control, striped pattern box) and NiVpp (white box) in the presence of 100 × diluted sera from different groups: (**a**) FCS and (**b**) normal bat sera (NBS), negative control sera; (**c**) rabbit anti-NiV, positive control; (**d**) bat sera from Cameroon (*n*=44). Seropositive (*n*=21, median bar in red) and seronegative (*n*=23, median bar in yellow) bat sera in **d** were segregated based on the Dunnett’s test for significance using the FCS control group. The boxes represent the first and the third quartiles, and the solid horizontal lines within the box represent the median values. The whiskers represent the lowest and highest value. Each sample was tested in quadruplicate. The data for each serum sample are shown in Supplementary Fig. 2a.

In our screen of bat serum samples (Fig. 2), ~48% (21/44) were classified as being positive for anti-NiV cross-neutralizing activity (Fig. 2d; Supplementary Fig. 2a) when compared against the fetal calf serum (FCS)-negative control group (Fig. 2a) as determined by the Dunnett’s test for multiple comparisons against a single control group. The specificity of our NiVpp SN assay is underscored by the lack of inhibition of the relevant serum samples against vesicular stomatitis virus-based pseudoparticles (VSVpp). Furthermore, ‘normal bat sera’ (NBS) from captive-bred bats in the United States did not show significant neutralization activity against NiVpp or VSVpp (Fig. 2b).

### Anti-NiV X-Nabs in human serum samples from Cameroon

The relatively high prevalence of anti-NiV-X-Nabs in the bat populations surrounding Yaoundé in southern Cameroon prompted us to examine archival *human* sera collected from this region of Africa for the presence of similar anti-NiV-X-Nabs, which might indicate potential spillover event(s). Thus, we analysed almost 500 blood samples collected from healthy adults by Global Viral/Metabiota in southern Cameroon between February 2001 and January 2003 in 13 different locations. All samples were collected in rural areas, but represent different habitats (savanna, gallery forest and lowland forest) supporting wild game populations that provided a source for the bushmeat trade (Supplementary Table 1).

**Figure 3:**
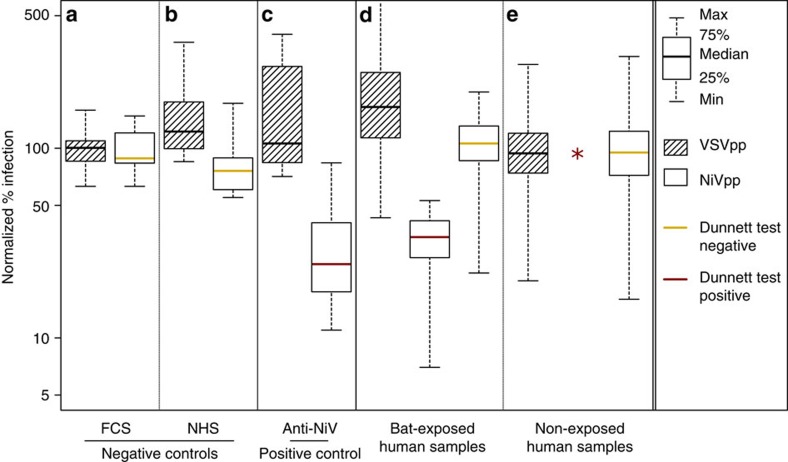
Seroneutralization activity of NiV pseudoparticle infection by human sera collected from Cameroon villagers with documented differential exposure to bats. Box-and-whisker plots showing the normalized % infection of Vero cells by VSVpp (isogenic control, striped pattern box) and NiVpp (white box) in the presence of sera diluted 1:100 from the indicated sample groups (Panels 1–5). (**a**) All infections were normalized to the infectivity observed for NiVpp in the presence of the fetal calf serum (FCS) negative control (set at 100%); (**b**) normal human sera (NHS) from Los Angeles blood donors served as additional negative controls; (**c**) hyperimmune rabbit anti-NiV sera (positive control); (**d**,**e**) human sera from the bat-exposed (*n*=227) or non-exposed (*n*=260) cohort of Cameroon villagers, respectively. For the bat-exposed group, the Dunnett’s test could stratify the NiVpp SN results into seropositive (*n*=7, median bar in red) and seronegative (*n*=220, median bar in yellow) subsets. In contrast, in the non-bat-exposed group, the Dunnett’s test could not identify any serum sample as being significantly different from the negative control group (FCS; **e**), asterisk indicates no seropositive samples). Boxes encompass the first and the third quartiles, and the solid horizontal lines within the boxes represent the median values. The whiskers represent the lowest and highest values in each sample group. The data for individual serum samples (*n*=487), each tested in quadruplicates, are shown in Supplementary Fig. 2b and c.

The careful curation of samples allowed us to segregate the sera into various dichotomous groups such as those who reported contacts with bats and those that did not (Table 1). Sera were analysed using the same SN assay and criteria as described for the bat serum analysis except that normal human sera (NHS) from blood donors were used as complementary negative controls to FCS (Fig. 3). Using the Dunnett’s test as a stringent measure of significance, 7 out of 227 samples (~3%) in the bat-exposed group were considered to have significant anti-NiVpp cross-neutralizing activity when compared against the FCS group (Fig. 3d, Table 1 and Supplementary Fig. 2c and f). On the other hand, none of the 260 samples in the non-bat exposed group were statistically different from the FCS control group (Fig. 3e, Table 1 and Supplementary Fig. 2d and f). Seropositive samples exhibited different neutralizing potencies. One seropositive sample gave an IC_50_ titre of~1:1,000, whereas the other only achieved ~40% inhibition at 1:50 (the lowest serum dilution tested; Supplementary Fig. 2g). As expected, our hyperimmune anti-NiV control serum exhibited the highest IC_50_ titre of ~1:15,000. Interestingly, representative seronegative samples showed a slight enhancing effect as serum concentrations were increased, suggesting that we may even have underestimated the number of true positives.

### Specificity of anti-NiV X-Nabs in human serum samples

**Figure 4:**
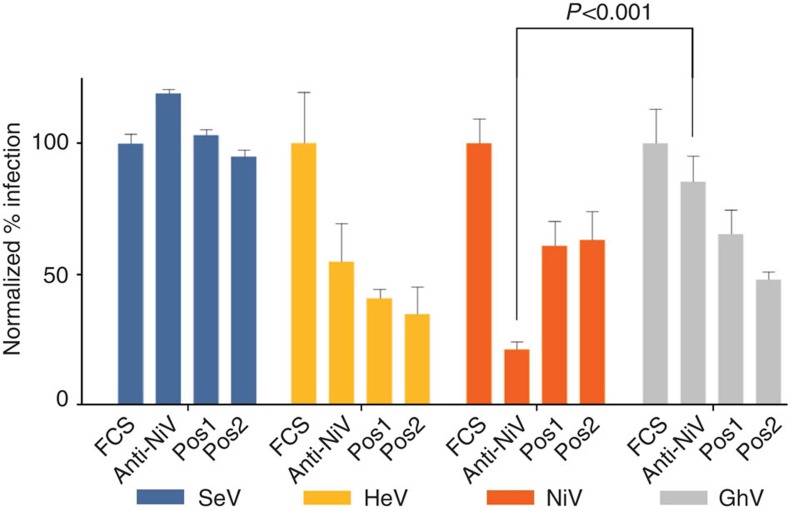
Characterization of seropositive human sera for anti-henipavirus cross-neutralizing antibodies. Two seropositive human samples (*Pos1* and *Pos2*) from the bat-exposed group were tested for seroneutralization activity against different HNV pseudoparticles: Hendra virus (HeV, yellow), Nipah virus (NiV, orange) and Ghana virus (GhV, grey). Infection of Vero cells was performed in the presence of the indicated sera diluted 1:100 as in Fig. 3. As a specificity control for the virus, we used a recombinant GFP-expressing Sendai virus GFP (SeV, blue). Data are presented as normalized % infection (mean±s.d. from three independent replicates) as in Fig. 3. FCS, fetal calf serum; anti-NiV, hyperimmune rabbit anti-NiV serum. Significant differences (asterisks) of inhibition was observed between the NiVpp and GhVpp for the anti-NiV serum (two-tailed Student’s *t*-test, *P*<0.001).

To further confirm the specificity of the human sera positive for anti-NiV-X-Nabs, and to determine the potential breadth of cross-reactivity of these antibodies, we chose two seropositive samples and tested them in our SN assay against three HNV pseudoparticles: NiVpp, HeVpp and GhVpp, the latter bearing F and G from the Gh-M74a clone reported by Drexler *et al.*^17^, and an enhanced green fluorescent protein (eGFP)-expressing recombinant Sendai virus (rSeV-eGFP). SeV is a murine paramyxovirus (*Respirovirus* genus) not normally found in humans that enter cells via a pH-independent pathway using sialic acid-based receptors, and thus, should not be enhanced or inhibited by the seropositive human samples identified in Fig. 3. Indeed, neither the seropositive human Cameroon sera nor the hyperimmune rabbit anti-NiV serum inhibited the pH-independent entry of rSeV-eGFP (Fig. 4, blue bars). In contrast, seropositive human Cameroon samples inhibited NiVpp, HeVpp and GhVpp to varying degrees (Fig. 4, red, orange and grey bars, respectively). Interestingly, our hyperimmune anti-NiV sera showed good to moderate (50–80%) inhibition of NiVpp and HeVpp infection but only minimal inhibition (~10–15%) of GhVpp infection. The implications of these results will be discussed.

As a final confirmation regarding the specificity of the anti-NiV-X-Nabs that we detected in the seropositive human samples, we repeated the SN assay with live NiV under BSL-4 conditions. In order to conserve the use of these archival sera, which were only available in small amounts, we generated and rescued a recombinant NiV expressing secreted *Gaussia* Luciferase (rNiV-GLuc; see Methods and Supplementary Fig. 3a). *In vitro* growth kinetics of rNiV-GLuc was comparable to the parental NiV Malaysian strain and pathogenicity was demonstrated in the hamster model where bioluminescence in *ex vivo* harvested organs from the moribund animal correlated well with viral titres measured by traditional plaque assays (Supplementary Fig. 3b). Furthermore, when we infected Vero cells with rNiV-GLuc across a broad range of multiplicity of infection (MOI) (0.01–3), GLuc activity in cell culture supernatant at 24 h.p.i. was significantly and positively correlated with viral titres determined by traditional plaque assays (Supplementary Fig. 4A, *r*^*2*^=0.93, *P*=0.008). Thus, rNiV-GLuc allowed us a highly sensitive and dynamic method to monitor NiV infection (or inhibition thereof) in cell culture by sampling infected cell culture supernatant for *Gaussia* luciferase activity. We then chose four seropositive and two seronegative human samples from our Cameroon cohort, and performed our SN assay with rNiV-GLuc along with the other appropriate negative and positive controls.

**Figure 5:**
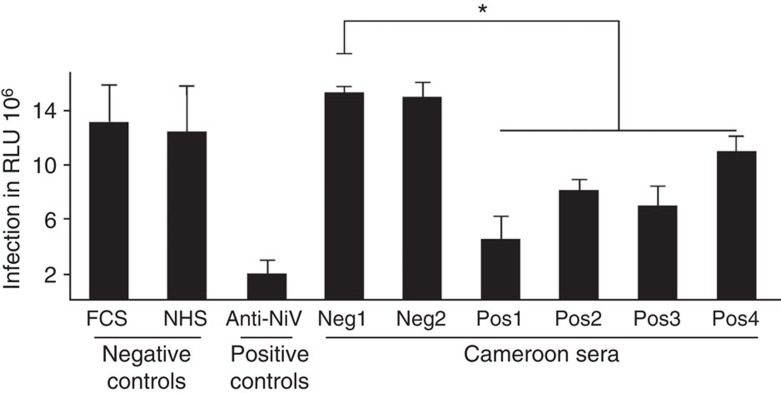
Seropositive human samples neutralize live recombinant NiV infection. Infection of Vero cells by live recombinant NiV expressing secreted *Gaussia* luciferase (rNiV-GLuc, MOI=1) in the presence of 1:100 dilution of the indicated sera: FCS (fetal calf serum, negative control), NHS (normal human serum, negative control), rabbit anti-NiV (positive control), two seronegative samples (picked randomly among the 480 seronegative samples) and four seropositive samples. 20 μl (out of 150 μl) of infected cell culture supernatant was collected and analysed for *Gaussia* luciferase activity at 24 h.p.i. Infectivity data are presented as mean relative light units (RLUs)±s.d. from three independent replicates. Significant differences (asterisk) were observed between seropositive and seronegative sera (*P*<0.05; one-tailed Student’s *t*-test followed by the Holm step-down procedure for multiple comparisons).

Figure 5 shows that only the seropositive samples significantly inhibited rNiV-GLuc infection, albeit with varying degrees of potency (15–65% reduction in GLuc activity). A relatively high inoculum was used, and raw luciferase activity values (>10^7^ relative light units (RLUs)) are presented to demonstrate the robustness of our assay. Supplementary Fig. 4B and C shows how inhibition of GLuc activity correlated with decreases in viral titres across a range of MOI, and also indicates that we used a high MOI inoculum (≥3). Seronegative samples showed no significant inhibition of rNiV-GLuc infection, and the GLuc activity detected was not different from the FCS-negative or NHS-negative controls. As expected, the hyperimmune rabbit anti-NiV serum inhibited rNiV-GLuc infection by close to 90%.

### Risk factor analysis

The questionnaire filled out by the participants before blood sample collection covered their contacts with some animals known to be HNV hosts, some of their ‘at risk’ activities and the location of the village with its associated environmental features. We analysed their answers to uncover any risk factors that might be associated with seropositivity. Table 1 shows that all of the seropositive samples came from the group that reported contact with bats in one form or another with those exposed to bats being 17 times more likely to be HNV seropositive (odds ratio=17.72, *P*=0.0021, two-tailed Fisher’s Exact test with zero-cell correction^24^,^25^). The highly statistically significant difference between seroprevalence rates in the bat-exposed (7/227, 3%) versus non-exposed groups (0/260; *P*=0.0045, Fisher’s Exact test) supports the hypothesis that contact with bats increases one’s risk of being infected by an antigenically related African HNV-like virus.

We next tried to determine whether a particular type of bat exposure was more significantly associated with seropositivity. The detailed questionnaire allowed us to segregate the tested serum samples into other dichotomous groups such as those who butchered bats (or not), those who hunted bats (or not) and those who remembered bites/scratches from bats (or not). Intriguingly, hunting bats alone was not a sufficient risk factor; however, those butchering bats were 29 times more likely to be seropositive than those not having contact with bats (7/164 (4%) versus 0/316, respectively; *P*=0.0002; Table 1). Although there was no statistically significant association with gender, it is interesting to note that with the exception of one 85-year-old case there were no seropositives above the age of 45 years (Supplementary Fig. 5).

**Figure 6:**
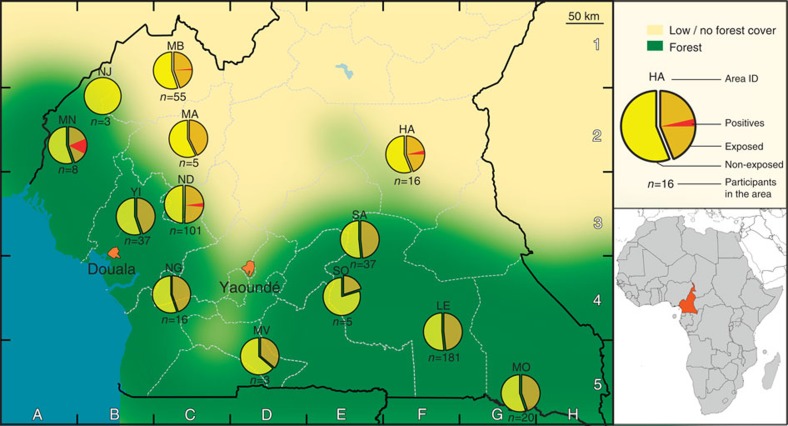
Map of collection sites in Southern Cameroon. For each location, the proportion of participants with self-reported contacts with bats (exposed) or not (non-exposed) is indicated, respectively, by the brown or yellow segment of the accompanying pie-chart. The superimposed red segments in some pie-charts represent the seropositive samples identified in Fig. 4 (also Supplementary Fig. 2b). More detailed information on the samples collected from each region is presented in Supplementary Table 1. The number of participants from each location is shown below the corresponding pie chart. Green- or beige-shaded areas represent regions with high or low/no forest cover, respectively (see ‘Geographical data’ section in Methods for further details).

Finally, we examined environmental and geographic parameters. Figure 6 shows a map of the indicated areas of our sample collection sites, and Supplementary Table 1 lists the map coordinates of the indicated areas shown in Fig. 6, for example, ND is in grid C3. The majority of the seropositive participants were from the ND area (4/7), whereas the others were from MB (C1), MN (A2) and HA (F2) areas. Ground reporting from Global Viral/Metabiota staff at the time of collection revealed that seropositive samples came almost invariably from low forest cover areas (Fig. 6). Indeed, Table 1 shows that seroprevalence for HNV-like viruses were significantly higher in samples originating from these four areas compared with those that did not (3.2% versus 0.3%, respectively, *P*=0.0136). In effect, those living in areas of putative deforestation were ten times more likely to be HNV-seropositive than those who were not (odds ratio=10.1, *P*=0.0088). In addition to the seven ‘true’ seropositive samples classified by the Dunnett’s test, there were three borderline-positive samples (~50% inhibition) in the bat-exposed group (Supplementary Fig. 2e, blue) that also came from areas associated with deforestation: ND, MN and HA (located in C3, A2 and F3, respectively, in Fig. 6 map). Within the ND area, 3 of the 4 seropositive samples were from the same village, and only 12 persons from this village with known bat contacts volunteered for this study, which would have resulted in a 25% seroprevalence rate among the bat-exposed group if this village were considered alone.

## Discussion

In this study, we provide multiple lines of evidence that suggest HNV-like spillover events from its natural bat reservoir into the human population in southern Cameroon. Using our optimized pseudotyped virus SN assay as a primary screen for almost 500 human serum samples, we confidently identified at least seven HNV-seropositive samples based on stringent statistical criteria, internal specificity controls for both the virus and the sera, and follow-up confirmation with live recombinant NiV (rNiV-GLuc) or SeV, a paramyxovirus from a different genus. Together, these precautions enabled the risk factor analysis that revealed highly significant associations of seropositivity with the behavioural and environmental parameters that are known to facilitate zoonotic emergence^26^,^27^,^28^,^29^.

By combining behavioural, geographic and serological data, we can provide more conclusive results than that which would be available from inclusion of one set of data alone. For example, that seropositivity was exclusively and most significantly associated with intimate bat exposure (*P*=0.0006), such as the slaughtering of bats for bushmeat (Table 1), is more informative than results from a SN assay with perfect sensitivity and specificity unlinked to an exposure risk. As for environmental factors, all seropositive samples come from villages located near open savannah lands or areas of deforestation as documented by our local field staff. *In toto*, we provide a strong body of evidence indicating spillover of HNV-like viruses into the human population in Africa.

In recent years, several groups have detected HNV-like sequences in African wildlife (bat) and domesticated pig populations^10^,^14^,^15^,^16^,^17^. A more recent study^22^ provides evidence for continent-wide panmixia of the HNV reservoir host, the common straw-coloured African fruit bat (*E. helvum*), and reports an average seroprevalance of ~42% for cross-reactive NiV-G-binding antibodies using the Luminex assay. This is close to the 48% seroprevalance rate we found in our cohort of *E. helvum* serum samples using our SN assay. However, the former study reported a much lower seroprevalance rate (~5% in Tanzania and ~15% in Ghana) when using their Luminex-based serum antibody/sEphrinB2 competition assay as a surrogate viral neutralization test. These contrasting results suggest that our infectious NiVpp SN assay has increased sensitivity, perhaps due to the ability of our SN assay to detect neutralizing anti-F antibodies as well as neutralizing anti-G antibodies that do not compete for sEphrinB2 binding^30^.

The ability of seropositive bat sera to cross-neutralize NiVpp infection suggest a close antigenic relationship between the envelope glycoproteins of NiV and that of the putative ‘Cameroon’ HNV strain(s). Drexler *et al.* also showed that serum from the African bat that was infected with the parental GhV (Gh-M74a) exhibited cross-reactivity with antigens expressed on NiV-infected cells^17^. Mapping the sequence of GhV-G onto the NiV-G crystal structure indicated that despite the low overall sequence identity (~25%) between the two attachment glycoproteins, the ephrinB2 receptor-binding site was relatively conserved suggesting common receptor usage between these two divergent HNVs (Fig. 1). Indeed, sEphrinB2-Fc inhibits GhVpp infection, binds to cell surface-expressed GhV-G^31^,^32^ and immunoprecipitates GhV-G^33^, suggesting that GhV is a related African HNV that also uses ephrin B2 as an entry receptor. Conservation of receptor usage is strong biological evidence that GhV is a bona fide HNV, albeit distantly related to NiV and HeV. Indeed, the ability of a virus to use highly conserved receptors has predictive value when considering the likelihood of viral emergence and cross-species spillover^28^,^34^.

The results of our SN assay comparing seropositive Cameroon human sera samples with our hyperimmune rabbit anti-NiV serum (Fig. 4) suggest that GhV, or at least the GhV-F and/or -G, is antigenically closer to the putative HNV common ancestor than NiV or HeV. Antibodies made against a more ‘ancestral’ virus such as the presumptive African HNV-like virus that infected the seropositive individuals, have a greater breadth of cross-neutralization than antibodies made against a more divergent lineage isolated by genetic drift (for example, anti-NiV), which does not neutralize GhVpp well, if at all. Note that the putative ‘Cameroon’ HNV strains that infected and elicited the anti-NiV-X-Nabs from the seropositive individuals were not even likely to be the same strain as GhV itself, and yet elicited antibodies that cross-neutralized NiV, HeV and GhV. These results have implications for a broad-coverage vaccine strategy: the use of envelope glycoproteins from a more ‘ancestral’ African HNV clade could induce more potent cross-neutralizing antibodies against emerging HNV-like viral strains. The increased breadth of cross-neutralization elicited by more ancestral viral envelopes have already been documented for HIV^35^ and influenza virus^36^, and forms the basis of vaccine strategies to elicit broadly neutralizing antibodies.

Given the relative ubiquity of HNVs or HNV-like viral agents in Africa, the commonality of the bushmeat trade in the resource-poor areas under study, and the presence of HNV-like sequences in up to one-third of *E. helvum* sold as bushmeat in neighbouring Brazzaville, Congo^16^, we surmised that the conditions were alarmingly optimal for zoonotic transmission of African HNVs from a bat reservoir host to the human population group at highest risk for such zoonoses. If we classify all 487 serum samples by those that reported butchering bats (*n*=171) and those that did not (*n*=316), the seroprevalence rate for anti-NiV-X-Nabs among bat butchers is ~4% (7/171, Table 1). As a reference point, HIV-1 prevalence in Cameroon was estimated to be approximately 5% by UNAIDS in 2010.

Although no human HNV encephalitis cases have ever been documented in Africa, this does not preclude the existence of outbreaks or spillovers. Considering the shortage of physicians (1/10,400 according the WHO) and the endemicity of malaria, yellow fever, typhoid fever and meningococcal meningitis, it is not surprising that an emerging encephalitic virus would remain misdiagnosed and unreported. Indeed, HNV infections have a history of being misdiagnosed: NiV was initially incorrectly identified as Japanese encephalitis virus when it first appeared in 1998 (ref. 37) and the Siliguri outbreak of NiV was originally reported as ‘aberrant measles’^38^. It is also possible that some of these African HNV-like viruses are non-pathogenic, not unlike Cedar Virus, a non-pathogenic strain of HNV isolated from Australian bats^39^. In any case, the virulence of African HNV-like viruses awaits experimental confirmation. In all likelihood, the high diversity of HNV-like viruses in Africa suggests a virulence spectrum that is equally diverse.

As humans and/or their domesticated animals encroach upon the ecological niche occupied by the reservoir hosts, increased opportunities for contact with the virus reservoir also increases the risk for cross-species infection^40^,^41^. The destruction of natural habitats can also lead wildlife to relocate, sometimes within greater proximity to human populations^27^,^28^,^41^,^42^. Our field data indicate that the vast majority of seropositive participants come from the western part of the country (ND, MN, MB), where a lack of forest cover because of natural or human causes was a noted feature (C3, A2 and C1 in Fig. 6). The clustering of seropositive participants is particularly striking in a village of the ND area where 3 out of 12 (25%) participants were positive. These three participants are all young adults (25–35 years old) with documented bat butchering activities. On the other hand, none of the >200 samples from the deep tropical forest locations such as LE and MO (Fig. 6, F4 and G5, respectively) were positive even though ~50% of these samples came from the bat-exposed group.

Bat hunting in Cameroon—typically by use of firearm, nets or catapults—do not involve physical contact between bats and hunters. Consistent with these observations, hunting bats *per se* was not associated with an increased risk for seroconversion. Furthermore, none of the pig owners or hunters (that do not also butcher their catch) enrolled in our study were seropositive. Thus, superficial contact with domestic animals does not appear to increase the risk of a HNV-like infection. In contrast, that butchering bats is the most significantly associated risk factor for HNV seropositivity suggests that close contact with bodily fluids (blood, saliva, excreta) is likely required for successful cross-species transmission.

Until recently, the range of the reservoir hosts was thought to confine HNV spillovers to Asia and Australia. However, there is increasing serological and molecular evidence documenting the widespread occurrence and diversity of HNVs in Africa, mainly in African fruit bat species. Our study now provides evidence for HNV spillover into human population groups in Africa (Cameroon) at high-risk for contracting zoonoses. In the various taxonomic schemes proposed for the transitional dynamics of zoonotic pathogens^27^,^43^, features such as the (i) prevalence and diversity of HNVs in their African reservoir hosts, (ii) the increased opportunities for zoonotic transmission provided by the bushmeat trade, (iii) the unusually broad species tropism of HNVs facilitated by the use of highly conserved receptors and (iv) the documented human-to-human transmissibility of NiV, justifiably place HNV or HNV-like viruses at or close to the penultimate stage for sustained transmission in human outbreaks. Our data warrant increased surveillance efforts to determine the frequency of similar spillover events in Africa at large, and highlights the need for international collaborations and cross-disciplinary approaches to determine the virulence spectrum of African HNVs.

## Methods

### Mapping the putative GhV-G-ephrin-binding interface

Based upon sequence similarity with the NiV (24%) and HeV (25%) attachment glycoproteins, the C-terminal 430 amino acids of GhV-G are predicted to comprise a globular six-bladed β-propeller domain^44^. To predict if the GhV-G β-propeller also shares receptor-binding specificity for ephrinB2 and ephrinB3, sequence conservation between GhV-G and NiV-G was mapped onto the crystal structure of NiV-G in complex with ephrinB2 (PDB accession code 2VSM)^23^. NiV-G residues involved in ephrin binding were identified with the PISA EBI server^45^ and a structure-based sequence alignment of NiV-G, HeV-G and GhV-G was calculated with ClustalW^46^ and plotted with ESPript^47^. Residue conservation mapping and image rendering was performed with the programme PyMOL (http:// www.pymol.org).

### Bat serum samples

Blood samples were obtained from dead, wild *E. helvum* fruit bats (*n*=45) hunted by local hunters in Yaoundé, Cameroon, between 8 May 2004 and 9 June 2007 in accordance with approvals from the Cameroon Government and Johns Hopkins University IACUC approvals (FS03M221 and FS06H205). No payments were made in relation to the collection of samples to ensure no increased hunting of bats occurred as a result of this research. Dead bats were bled by cardiac puncture shortly after death with a 3 ml syringe. The blood was transferred to EDTA (plasma) or CAT Plus (serum) vaccutainer and centrifuged at 300*g*/1,300 r.p.m. for 15 min. NBS samples were obtained fr*om Pteropus hypomela*nus that were born and raised in captivity in the United States (a kind gift from the Brevard Zoo, Melbourne, Florida, USA). Serum samples sent to UCLA were leftovers from a routine check-up of the animals in Fall 2011, and were considered as discarded material and exempt from IACUC approval by the veterinary team at Brevard Zoo. They were not collected prospectively or specifically for this project.

### Human serum samples

Participation in the study was voluntary. Description of the study, informed consent procedures and questionnaire administration were done orally in either French or English, which are widely spoken as second languages in study villages. Participants were offered compensation approximately equivalent to 1 day of work, as participation precluded farm work on that day. The study protocol was approved by the Johns Hopkins Committee for Human Research, the Cameroon National Ethics Committee and the HIV Tri-Services Secondary Review Board. In addition, a single project assurance was obtained from the Cameroonian Ministry of Health and accepted by the National Institutes of Health Office for Protection from Research Risks. The UCLA Internal Research Board (IRB) confirmed that the use of these anonymized archival serum samples did not constitute ‘human subjects’ research and thus no independent IRB review was required. Human blood samples were collected on site by the Global Viral/Metabiota (previously known as GVFI) team from 497 participants between 2001 and 2003 in 13 different areas around southern Cameroon. Once drawn, the blood was transferred to EDTA (plasma) or CAT Plus (serum) vaccutainer and centrifuged at 300*g*/1,300 r.p.m. for 15 min. Sera were stored at −80 °C until processing for utilization in assays.

### Serum sample handling and preparation

All bat and human serum samples were handled according to proposed WHO guidelines for working safely with diagnostic field specimens^19^. Sera were first heat-inactivated at 56 °C for 30 min, and then treated with Triton X-100 under BSL-2 conditions to ensure pathogen inactivation. All procedures were approved by the UCLA Institutional Biosafety Committee.

### Vesicular stomatitis virus-based pseudoparticles

VSVpp were produced following established protocols^20^. Briefly, recombinant VSV with a *Renilla* luciferase reporter gene engineered in place of its native envelope glycoprotein (VSV-ΔG-rLuc) was pseudotyped with either its own G protein (**VSV-Gpp**), or the F and G envelope glycoproteins of NiV (**NiVpp**; Genbank accession codes NC_002728.1, GI:13559808), HeV (**HeVpp**; Genbank accession codes: NC_001906.3, GI:529283690), or the newly described African HNV from Ghana (**GhVpp**; clone Gh-M74a^17^, Genbank accession codes: HQ660129.1, GI:384476032). Pseudotyping was accomplished by transfecting 293T cells with codon-optimized expression plasmids for the F and G envelope glycoproteins of NiV, HeV, and GhV, or for the VSV-G glycoprotein itself, and then infecting with VSV-ΔG-rLuc (complemented with VSV-G). Twenty-four hours after infection, pseudotype-containing media were clarified of cell debris by centrifugation at 1,500 r.p.m. for 5 min. Supernatants were then loaded on a 20% sucrose cushion and ultra-centrifugated for 2 h at 110,000*g*. The pellet of concentrated pseudoparticles was then resuspended in Opti-MEM (Life Technologies), aliquoted and stored at −80 °C.

### GhV-F sequence rectification

Sequence inspection and bioinformatics analysis indicated that the GhV-F sequence in Genbank (accession code AFH96010.1) is likely incorrect due to a single-nucleotide deletion near the N-terminus, which resulted in an extra-long N-terminus with no predicted signal peptide. The details, rationale and functional evidence for sequence rectification of the GhV-F gene are provided elsewhere^32^.

### Recombinant Sendai virus and Nipah virus

The rSeV is a modified version of RGV0 (a kind gift of Nancy McQueen), a Fushimi strain construct with F1-R strain mutations in F and M as described by Hou *et al.*^48^ We inserted an eGFP reporter between the N and P genes and made further modifications to increase rescue efficiency^49^.

Recombinant NiV (reference Malaysian strain, Genbank accession codes NC_002728.1, GI:13559808), rNiV-GLuc, was engineered to express secreted *Gaussia* luciferase (descriptive name: NiV_MAL_ T7_P-3G_ 3'Ribozyme A-(N_GLuc-p2A-eGFP_P) as described by Yun *et al.*^50^). The *Gaussia* luciferase (GLuc) open reading frame was modified with two mutations that provide greater signal and stability, M60L and M127L (refs 51, 52); these residues are called M43 and M110 in refs 51, 52 due to removal of the 17-aa secretion signal peptide. Rescue of rNiV-GLuc and SN assays were performed at the UTMB Galveston National Laboratory BSL-4 laboratory.

### Optimization of VSVpp SN assay

Using a reference panel of human and pig sera, we and our collaborators at the United States Centers for Disease Control, Canadian Food Inspection Agency and Merial Sanofi (Lyon, France), previously validated our NiVpp SN assay to have a specificity of 94–100%, and an equivalent or lower sensitivity when measured against a standard live NiV plaque reduction neutralization test as the gold standard^20^.

For our current study, we first determined the linear dynamic range of each pseudoparticle preparation, and a fixed amount of virus within the linear range (corresponding to the luciferase reporter output of ~20,000 RLU at 24 h.p.i.) was chosen for subsequent SN assays (Supplementary Fig. 1a). Next, the optimal serum dilution to be used was determined by comparing the SN activity of a well-characterized hyperimmune rabbit sera made against Nipah virus-like particles bearing both the NiV fusion (F) and attachment (G) envelope glycoproteins^53^, and its pre-immune counterpart. Significant differences were observed for dilutions between 1:100 and 1:10,000 (Supplementary Fig. 1b). Based on these data, we diluted bat and human sera 1:100 for all our SN assays. Use of high serum dilutions (>1:20) might also mitigate putative serum-induced cytotoxicity effects that often occur at high serum concentrations, which can confound SN results^54^,^55^. As an additional specificity control, we used an isogenic VSV-ΔG-rLuc pseudoparticle containing the envelope glycoprotein of VSV itself (Fig. 2 and Supplementary Fig. 2). VSV is endemic to the Americas, and has not been reported in Africa since 1900 (refs 56, 57, 58, 59), so any serum samples that show inhibition of both VSVpp and NiVpp infection was considered nonspecific or cytotoxic, and was discarded from further analysis. In all, 1 out of 45 and 10 out of 497 bat and human samples, respectively, strongly inhibited both VSVpp and NiVpp infection and were discarded from analysis.

For the valid samples, SN assays were performed in DMEM (Invitrogen) containing 1:100 dilution of sera and an optimized amount of pseudotyped virus inoculum (VSVpp, NiVpp, HeVpp, or GhVpp) that will result in ~20,000 RLU of luciferase activity at 24 h.p.i. The medium containing infectious virus and serum was transferred to a monolayer of Vero cells and incubated at 37 °C for 2 h before removal and replacement with fresh DMEM containing 10% FCS. Cells were incubated at 37 °C for another 24 h before processing for detection of *Renilla* luciferase activity according to the manufacturer’s directions (Promega). SN titres were performed using identical procedures except that the viral inoculum (NiVpp) was pre-mixed with serial fivefold dilution of sera from 1:50 to 1:31,250. All infections were performed in quadruplicates.

### Live virus SN assay

The relevant bat and human serum samples were also tested for neutralizing antibodies using the replication-competent recombinant paramyxoviruses (rSeV-eGFP or rNiV-GLuc) generated as described above. SN of live rSeV-eGFP and rNiV-GLuc infection was performed in an identical manner as VSVpp (the latter under BSL-4 conditions), except that rSeV-eGFP infection was detected by FACS analysis and rNiV-GLuc infection was detected by quantifying GLuc activity (BioLux *Gaussia* Luciferase Assay, New England Biolabs) in 10% (v/v) of infected cell culture supernatant at 24 h.p.i.

FCS and hyperimmune rabbit anti-NiV serum^53^ were used as negative and positive controls, respectively. Additional negative controls included NHS from Los Angeles blood donors. These anonymized and de-identified blood samples were obtained on a fee-for-service basis from the Virology Core at the UCLA AIDS Institute. Core services were approved by and consistent with all IRB policies at UCLA. NBS from captive-bred bats were generously provided by Brevard Zoo, Melbourne, Florida, USA. All SN assays were performed in quadruplicates.

### Geographical data

Raw geographical data were extracted from http:// www.openstreetmap.org (OpenStreetMap contributors) and are available under the Open Database License ( http://www.openstreetmap.org/copyright). Maps were then built and modified with JOSM, Merkaartor and Inkscape software. Forest cover and deforestation were determined by onsite collaborators and documented in field reports.

### Statistical methods

SN assay results for quadruplicates were grouped for statistical analysis. Tests between groups were done using Dunnett’s test, a multiple comparison procedure for testing groups against a single control. Categorical data were tested and confidence intervals were estimated using Fisher’s Exact test. The strengths of association of seropositivity with bat contact and butchering were also estimated using two-tailed Fisher’s exact test. Because no exposure was observed in the unexposed, a value of 0.5 was added to all cells to allow the odds ratios to be calculated^24^,^25^. Statistical tests were performed using R version 2.15.1 for Mac and version 3.0.1 for GNU/Linux with the *multcomp* package. A modified R script was written to allow for use of non-integers in the Fisher’s Exact test.

## Additional information

**How to cite this article**: Pernet, O. *et al.* Evidence for henipavirus spillover into human populations in Africa. *Nat. Commun.* 5:5342 doi: 10.1038/ncomms6342 (2014).

## Supplementary Material

Supplementary Information Supplementary Figures 1-5, Supplementary Table 1 and Supplementary References

## Figures and Tables

**Table 1:** Risk factor analysis.

**Risk factor**	**Total**	**(** ***n*** **)**	**Seronegative (** ***n*** **)**	**Seropositive (*****n***)	**Seroprevalence (%)**	***P*** value*	**Odds ratio**† **(95% CI)**	***P*** **value**
Contact with bats	Yes	227	220	7	3.1	**0.0045**	**17.72**	**0.0021**
	No	260	260	0	0			
	Total	487					(1.01–312.02)	
Butchering bats	Yes	171	164	7	4.1	**0.0006**	28.86	**0.0002**
	No	316	316	0	0			
	Total	487					(1.64–508.45)	
Hunting bats	Yes	99	96	3	3.0	0.1523	3.00	0.1357
	No	388	384	4	1.0			
	Total	487					(0.66–13.63)	
Deforestation	Yes	185	179	6	3.2	**0.0136**	10.09	**0.0088**
	No	302	301	1	0.33			
	Total	487					(1.20–84.48)	

CI, confidence interval.

^*^Two-tailed Fisher’s Exact test.

^†^0.5 added to all cells where there is a zero cell count, see statistical methods. Bold entries indicate significant statistical differences.
