# Supplementary material for: Evidence for henipavirus spillover into human populations in Africa
Source: Nat Commun. 2014 Nov 18;5:5342. doi: 10.1038/ncomms6342 (PMC4237230; doi:10.1038/ncomms6342)
Supplement: Supplementary Information — Supplementary Figures 1-5, Supplementary Table 1 and Supplementary References [file ncomms6342-s1.pdf]

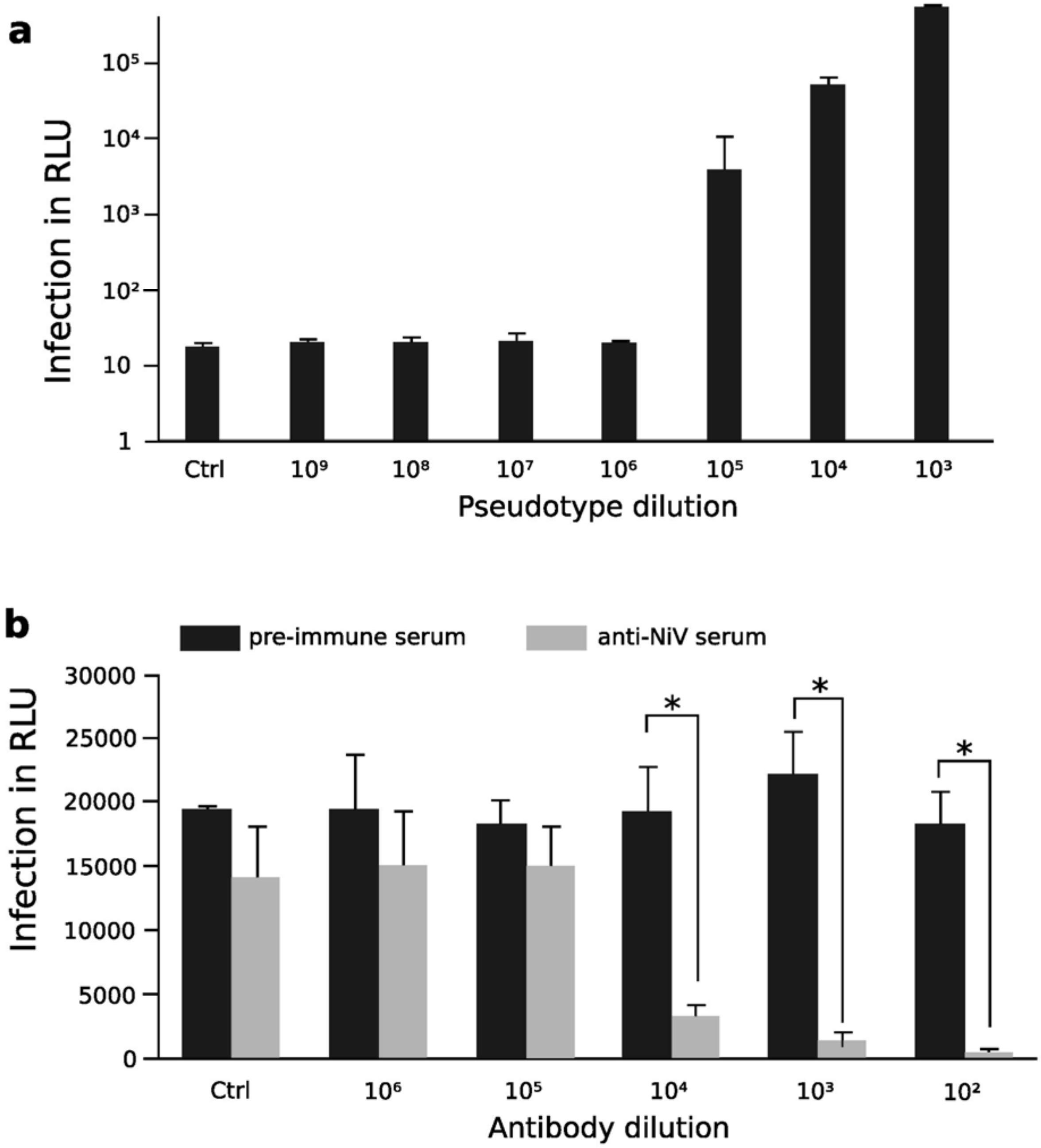

Supplementary Figure 1 | Optimization of the seroneutralization assay. **(a)** Determination of the linear dynamic range of VSV-ΔG-rLuc reporter NiV pseudotype particle (NiVpp) infection: Vero cells were seeded in a 96-well plate and infected with 10-fold serial dilutions of a NiVpp preparation (10^{-3} to 10^{-9}). At 24 hours post infection (hpi), cells were lysed and luciferase activity was quantified by luminometry as a measure of NiVpp

infection. Mock infection (Ctrl, infection medium only) gave negligible luciferase activity (RLU, relative light units) of less than 50 whereas a linear range of infectivity (~20,000 to ~200,000 RLU) was observed for viral dilutions between 10^{-3} and 10^{-5}. **(b)** Determination of the optimal serum dilution. Vero cells were seeded in a 96-well plate and infected with NiVpp in the presence of serial 10-fold dilutions (10^{-2} to 10^{-6}) of hyperimmune rabbit anti-NiV serum (grey) or its pre-immune counterpart (control, black). 24 hpi, cells were lysed and infection was measured by quantifying luciferase activity in the cell lysate as above. Significant differences (asterisks) between the two sera, indicating specific neutralization activity, were observed for serum dilutions from 10^{-2} to 10^{-4} ($p < 0.0002$; two-tailed Student t-test). Data are presented as mean +/- S.D. (n=4).

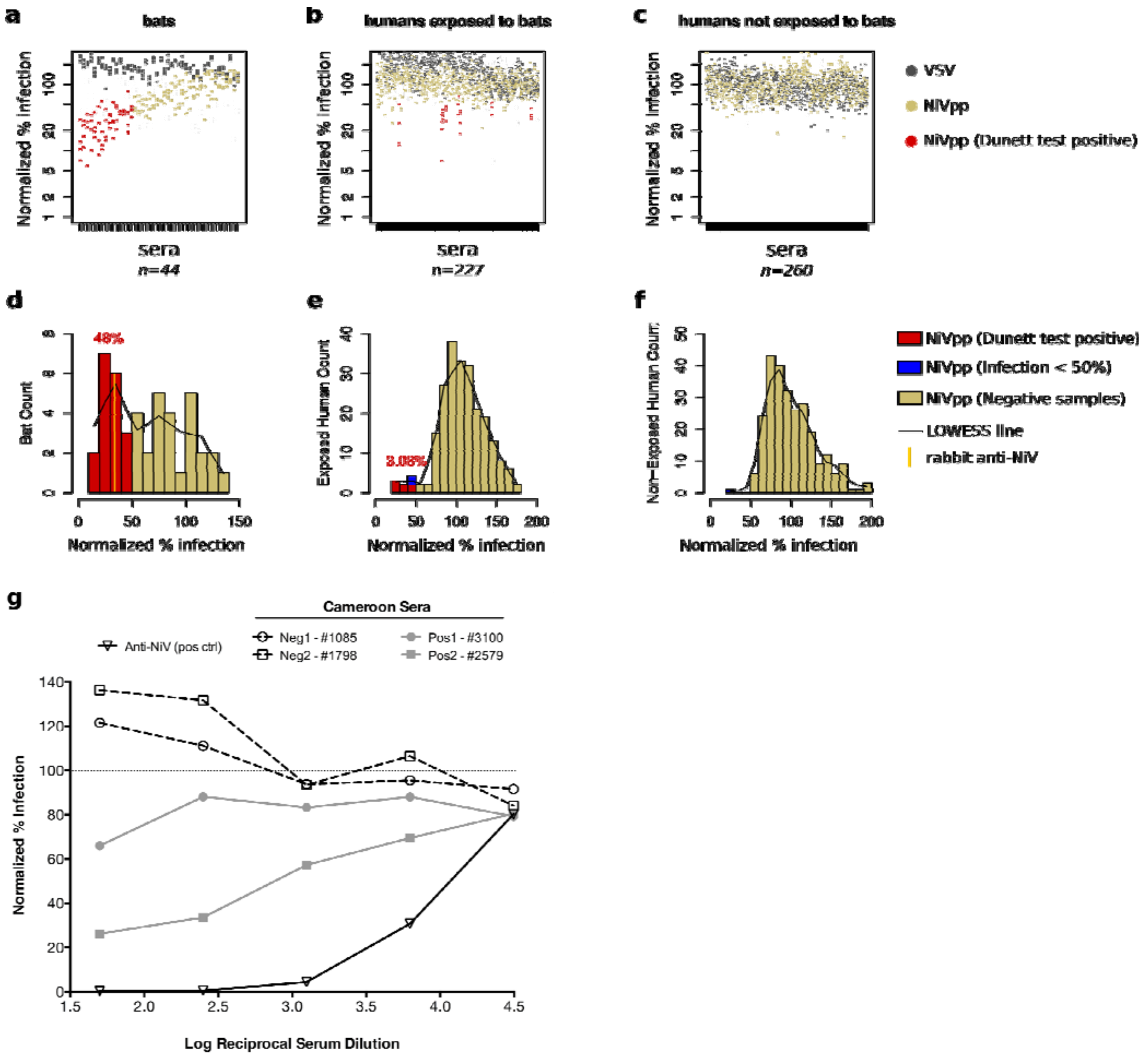

Supplementary Figure 2 | Primary data for individual serum samples screened with the infectious pseudotyped particle (VSVpp and NiVpp) seroneutralization assay. Dot plots representing the primary seroneutralization (SN) data for bat sera (**a**), bat-exposed human sera (**b**), and non-exposed human sera (**c**). SN data are presented as normalized % infection with infectivity obtained with NiVpp in the presence of FCS (1:100) set at 100%. Each sample, represented by the tick marks on the x-axis in panel (**a**), was tested in quadruplicate for NiVpp (gold or red dots) and VSVpp (black dots) inhibition. VSVpp is the isogenic virus control pseudotyped with VSV-G rather than NiV-F/G. Each vertical alignment of 8 dots (4 for NiVpp + 4 for VSVpp) represents the

results from a single serum sample. For NiVpp, seropositive samples, i.e., those classified as significantly different from the designated negative control group by the Dunnett's test, are indicated by red dots whereas the remaining seronegative samples are shown as gold dots. For the bat serum samples in panel (**a**), SN data are presented in order of their neutralization potency from left to right (sample with best SN activity is the first one on the left). For the bat-exposed (**b**) or non-exposed (**c**) human samples, SN data are presented in the order they were performed. The apparent enhancement of VSVpp infection seen with serum from the bat-exposed group (b), but not from the bat non-exposed group (c), is likely due to different shipment conditions: the former was exposed to long periods of dry-ice which resulted in carbon dioxide mediated acidification of the serum, thus enhancing entry by pH-dependent VSVpp, but having no such effect on pH-independent NiVpp entry. (**d-f**) The NiVpp SN data for the bat sera (**d**), bat-exposed human sera (**e**), and non-exposed human sera (**f**) were converted into frequency distribution histograms by grouping together samples in intervals of 10% of normalized infection. Seropositive and seronegative samples are indicated by red and gold bars, respectively. The LOWESS (Locally Weighted Scatterplot Smoothing**)** lines (in black) superimposed on the histograms identify two populations in panels (d) and (e), in agreement with the results from the Dunnett's test. The percentage of seropositive samples according to the Dunnett's test is indicated in red. In (b), serum samples (n=3) with ≥50% seroneutralization activity but not classified as seropositive by the stringent Dunnett's test appear in blue in panel (**e)**. Lastly, seroneutralization titers (SNTs) performed on representative seropositive and seronegative samples from the bat-exposed group are shown in **panel (g).** The SN assay was performed exactly as in (b) except that the viral inoculum was pre-mixed with 5-fold serial dilution of the indicated sera (1:50 to 1:31,250). SN data are presented as normalized % infection (mean values) with infectivity in 1:100 FCS set at 100% (dotted line) plotted against the log reciprocal serum dilution on the x-axis. Hyperimmune rabbit anti-NiV served as the positive control (inverted triangles). Infections were performed in quadruplicates. Mean values are shown without error bars for clarity.

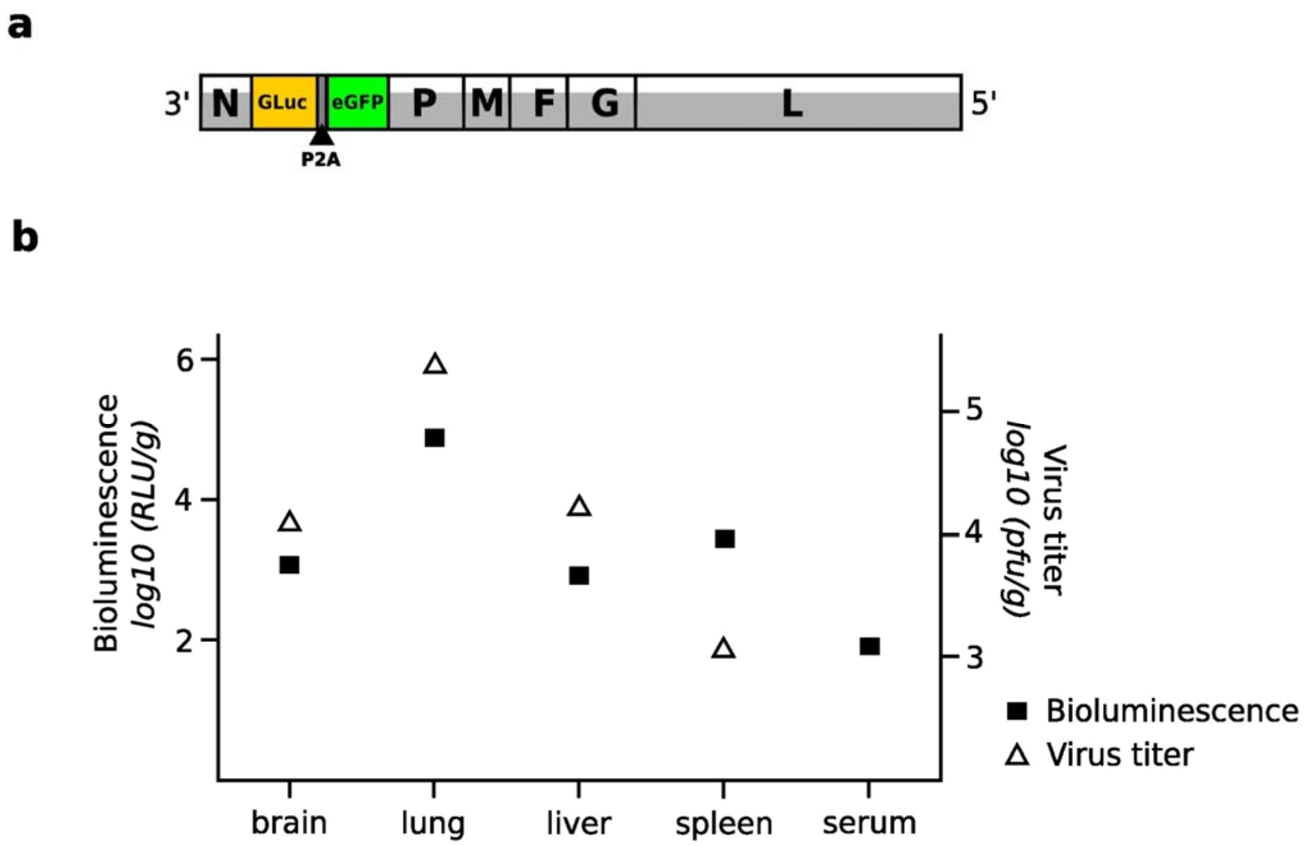

Supplementary Figure 3 | The recombinant NiV construct and the detection of secreted *Gaussia* Luciferase in different tissues. (**a**) Schematic representation of the reverse genetics construct used to rescue the recombinant NiV expressing eGFP and secreted Guassia Luciferase, which we term rNiV-GLuc. The GLuc-P2A-eGFP reporter cassette was inserted between a duplicated N-P intergenic region and the recombinant construct was rescued as described by Yun et *al.*[1]. Stocks of rNiV-GLuc were then expanded and tittered on Vero cells. (**b**) A hamster was challenged with 1,500 pfu of the rescued rNiV-GLuc virus (LD_{50} of NiV_{Mal} in hamsters ~250 pfu)[2] and the moribund animal was euthanized 6 days post-infection. Major organs (brain, liver, spleen, and lung) were collected and homogenized in PBS. GLuc activity was measured in clarified organ homogenates and presented as relative light units (RLU)/gram of tissue (or per ml of serum). Note that viremia is almost never detected in moribund animals as viral replication at that point occurs mostly in the brain and lungs[3]. Thus, the detection of secreted *Gaussia* luciferase activity in serum in the absence of detectable viral titers further underscores the sensitivity of this reporter for productive viral infection.

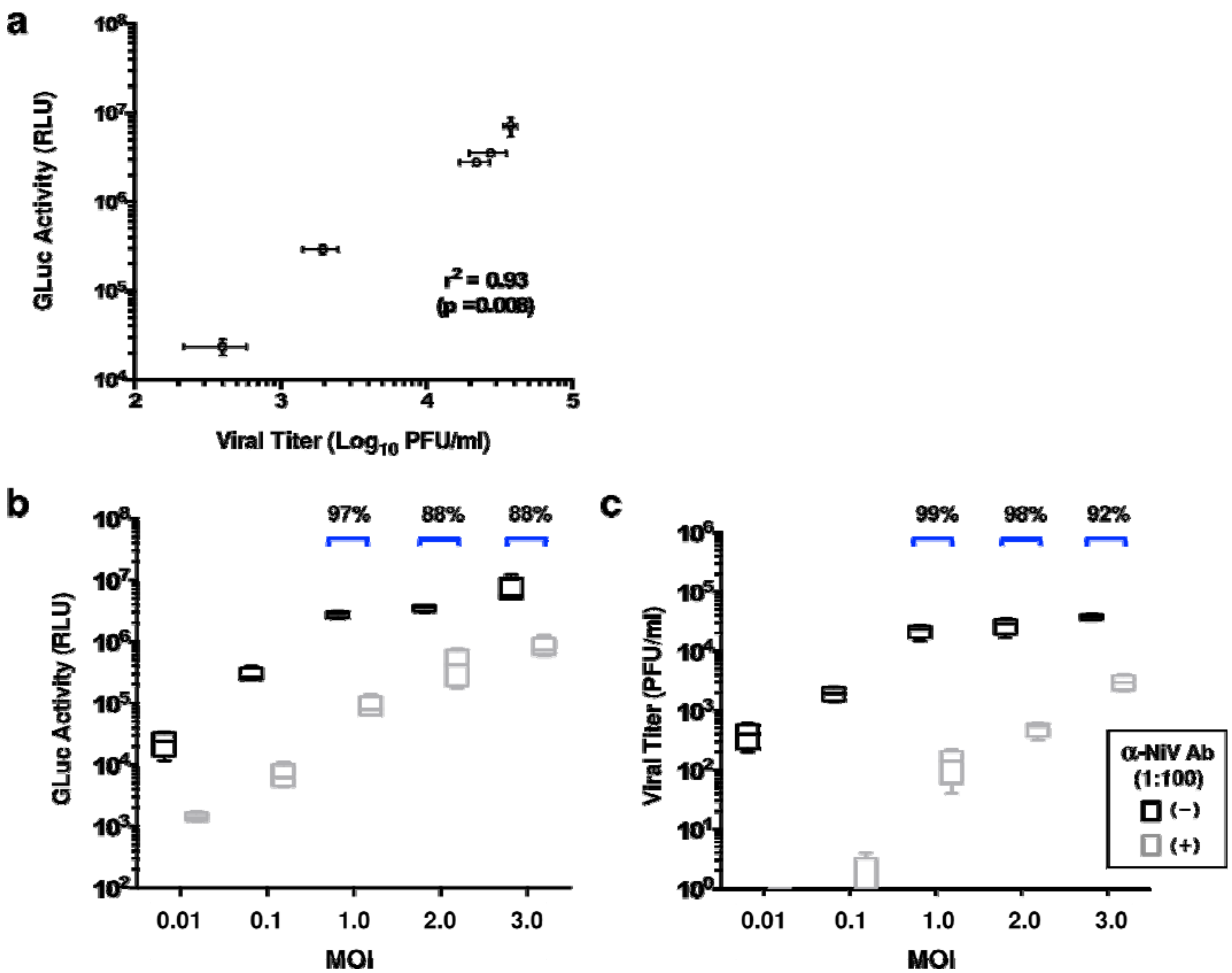

Supplementary Figure 4 | Correlation between the *Gaussia* Luciferase (GLuc) activity and virus titer using recombinant NiV-GLuc (rNiV-G-GLuc) reporter virus. Vero cells were seeded in a 96-well plate and infected at 5 different MOIs (0.01, 0.1, 1, 2, and 3) with live rNiV-GLuc, in the presence of 1:100 dilution of hyperimmune rabbit anti-NiV or control serum (Fetal Calf Serum, FCS). At 24 hours post infection, supernatant samples were taken: for each condition, GLuc activity was quantified by luminometry and titer was determined by traditional plaque assay. Mock infection (infection with medium only) gave negligible luciferase activity (RLU, relative light units) of less than 500 RLU and viral titers below our limit of detection (<10pfu) whereas a dynamic range of luciferase activity (~10^4 to ~10^7 RLU) corresponding to viral titers (>2 to <5 Log_{10} pfu/ml) were observed with live viral infections at different MOIs. **(a)** GLuc activity data are represented as mean RLU +/- S.D. from 4 replicates. Corresponding viral titers are represented as mean plaque forming unit (pfu) per mL +/- S.D from 4

replicates. Error bars are shown for both values at each data point. GLuc activity and viral titer showed highly significant (p=0.008) positive Pearson's correlation (r^2=0.93). **(b** and **c)** Box-and whiskers plots compared entry inhibition by neutralizing antibodies (grey boxes) to control serum (black boxes). Boxes represent the 5-95% range. For each MOI, a decrease in **(b)** GLuc activity corresponded to **(c)** an appropriate decrease of viral titer determined by traditional plaque assays. All conditions were tested in quadruplicate.

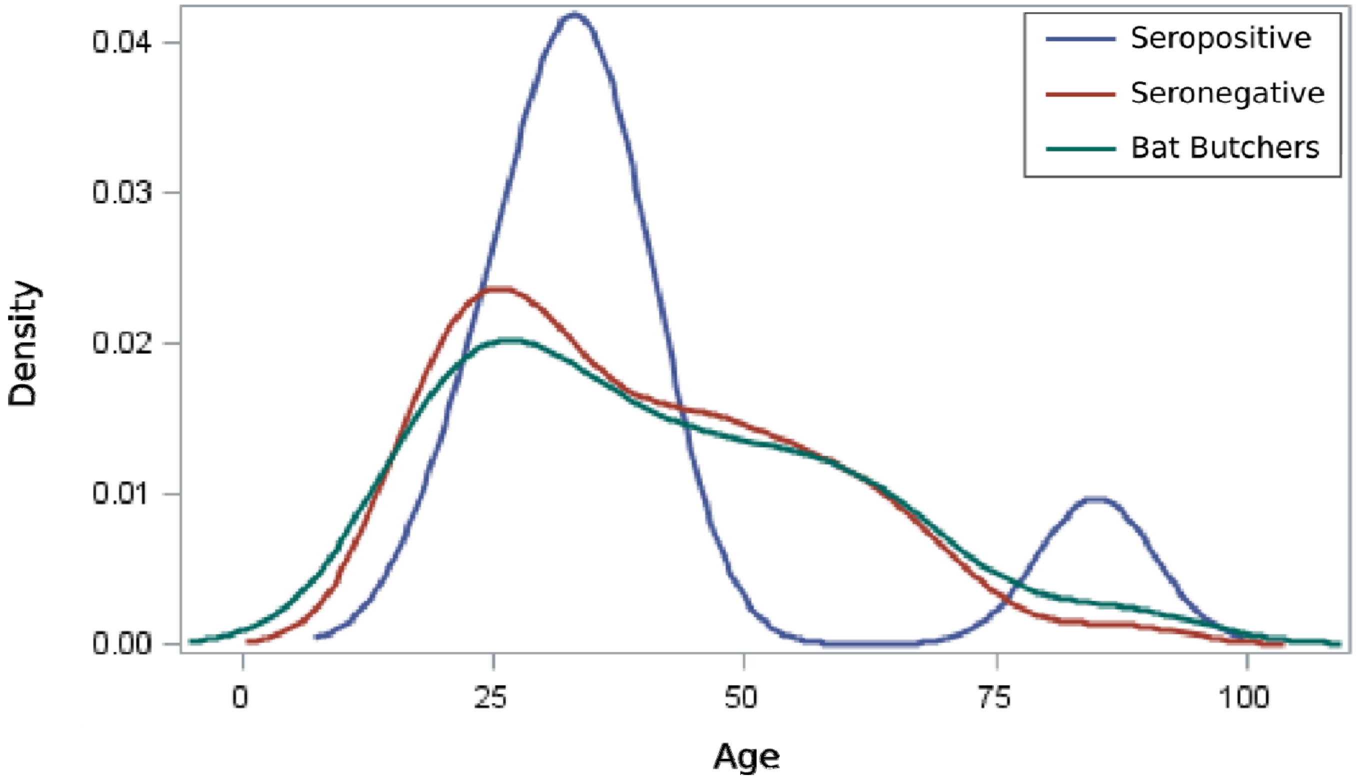

Supplementary Figure 5 | Age distribution of bat butchers grouped by their serostatus. The density (y-axis) is a kernel density estimator used to generate a smoothed histogram of age distribution (x-axis) from the raw data. The median age for seropositive (blue) and seronegative (red) bat butchers is 33 (IQR: 29-40) and 35 (IQR: 25-52), respectively. Note the lack of seropositives in the older age group (>45) with the exception of one 85-year old individual. The age distribution of bat butchers (green, n=171) taken as a group is similar to the age distribution of the seronegatives. The tight distribution of the seropositives (with the exception of the one 85-year old) suggests younger self-identified bat butchers may take on riskier roles (such as the physical slaughtering of bats). Future biosurveillance questionaires may require finer distinctions between what participants consider as "bat butchering" activities.

Supplementary Table 1. Area and location of human serum samples collected in Cameroon

Area	Location on the map	Non exposed	Bat exposed	Total	Non exposed Positive	Bat exposed Postive	Administrative Region name
HA	F2	9	7	16	0	1*	East
LE	F4	93	88	181	0	0	East
MA	C2	3	2	5	0	0	West
MB	C1	30	25	55	0	1	Northwest
MN	A2	5	3	8	0	1*	Southwest
MO	G5	11	9	20	0	0	East
MV	D5	2	1	3	0	0	Southwest
ND	C3	51	50	101	0	4*	Centre
NG	C4	10	6	16	0	0	Centre
NJ	B1	3	0	3	0	0	Southwest
SA	E3	19	18	37	0	0	East
SO	E4	4	1	5	0	0	East
YI	B3	20	17	37	0	0	Littoral
All		*260*	*227*	*487*	*0*	*7*	

References

1 Yun, T. E. *et al.* in *XV International Conference on Negative Strand Virus* 158 (Granada, Spain, 2013).

2 Wong, K. T. *et al.* A golden hamster model for human acute Nipah virus infection. *The American journal of pathology* **163**, 2127-2137, doi:10.1016/S0002-9440(10)63569-9 (2003).

3 Geisbert, T. W., Feldmann, H. & Broder, C. C. Animal challenge models of henipavirus infection and pathogenesis. *Current topics in microbiology and immunology* **359**, 153-177, doi:10.1007/82_2012_208 (2012).
